# The influence of flow velocity on coal fines output and coal permeability in the Fukang Block, southern Junggar Basin, China

**DOI:** 10.1038/s41598-017-14295-y

**Published:** 2017-10-26

**Authors:** Shu Tao, Dazhen Tang, Hao Xu, Song Li

**Affiliations:** 1 School of Energy Resources, China University of Geosciences (Beijing), Beijing, 100083 PR China; 2 Coal Reservoir Laboratory of National Engineering Research Center of CBM Development & Utilization, Beijing, 100083 PR China

## Abstract

Coal samples were cut into cores to perform a flow velocity sensitivity (FVS) analysis under varying initial permeability, confining pressures, injection volumes, and injection intensities. The results show that the permeability and the output of the coal fines decrease with an increase in confining pressures at a constant displacement velocity. A critical flow velocity exists for the migration of relatively large coal fines. Below this critical flow velocity, very small coal fines can be transported out of the coal by the fluid, slightly increasing the coal permeability. However, larger coal fines are transported at a higher flow velocity, which may block the effective seepage paths and reduce coal permeability, inducing FVS. Moreover, as the flow velocity and the injection volume increase, the permeability damage rate increases, but the rate of increase in the permeability damage decreases. The damage to the permeability due to FVS mainly occurs in the early stage of coal fines migration, and an abrupt increase in the flow velocity can damage reservoirs and induce substantial coal fines generation. Thus, maintaining a stable effective strength and a controlled depressurization rate during drainage can effectively constrain coal fines output and decrease permeability damage within coal reservoirs.

## Introduction

Coalbed methane (CBM) is a form of unconventional natural gas generated from coal and stored within the coal by an adsorption process. A coal reservoir has a dual-porosity structure, namely, a porous matrix surrounded by fractures^1,2^. Matrix porosity provides the main storage space for CBM, and fracture porosity is the main channel for gas flow. The former determines how much gas can be stored in the coal seam, whereas the latter determines how much gas can be discharged from the coal seam, which can also be characterized by permeability.

The permeability of a coal seam, a key parameter reflecting the ability of fluids to flow inside the seam, determines the migration and production of CBM. Many studies and production practices have shown that permeability is one of the main reservoir parameters controlling the extraction of CBM^3–5^. Due to the unique mechanical properties and gas production mechanisms of coal reservoirs, changes in the physical reservoir properties during the development process are different from those in conventional reservoirs. The dynamic change in permeability is the most significant physical property change^6,7^.

Drainage of CBM wells is a continuous process of discharging water, decreasing pressure, gas desorption, diffusion and seepage^7,8^. The permeability decline of a coal reservoir during the development of CBM may be caused by two main factors. The first factor is stress sensitivity. When the pore fluid pressure of the coalbed drops because of drainage from CBM production, the effective overburden pressure on the coalbed framework increases. Consequently, the reservoir will compress, and the coal permeability will be reduced due to the narrowing or even closing of the fracture apertures. The relationship between coal permeability and stress has drawn increasing attention in recent decades. Scholars have applied various methods to research the relationship between coal permeability and stress. These studies have concluded that coal reservoir permeability changes exponentially in relation to stress^9–12^. The second factor is flow velocity sensitivity (FVS)^13^. In reservoir formations, the change in the fluid flow velocity may induce coal fines migration and block pore throats, leading to the further decrease in reservoir permeability. Coal particles with a diameter of less than 0.3 mm are often produced along with coal seam gas and water, and these particles are called coal fines^14^. Coal is generally characterized by a small elastic modulus, low Poisson’s ratio, poor cementation and low hardness, suggesting that coal is prone to fracturing, which generates coal fines. During the development of CBM, the output of coal fines is inevitable due to mechanical collision, gas-liquid flow and pressure fluctuations, especially during fracturing stimulation^15–17^. Since coal fines have a low density and are strongly hydrophobic, they generally do not disperse within reservoir fluid and instead easily become concentrated. Gathered coal fines may plug coal microfractures and proppant-supported bed boundaries, reducing the permeability and flow conductivity of a coal reservoir^18^. The initial low permeability of coal may substantially decrease due to coal fines, affecting the production of CBM wells.

Coal fines have difficulty distributing in reservoir pores and will redistribute and migrate between reservoir pores as the flow velocity increases to a certain threshold. If the coal fines meet a flow channel with a small pore throat diameter, plugging will occur and decrease reservoir permeability^19–21^. Therefore, a study of coal fines may improve the efficiency of CBM development. However, only a few publications have addressed the FVS of coal reservoirs during CBM development.

In the current study, coal samples were collected from the Xishanyao Formation in the Fukang Block, southern Junggar Basin, China, and were used to conduct FVS experiments under various conditions. The relationship between the permeability change and fluid velocity was analyzed with the following purposes: (1) to determine the critical flow velocity for the occurrence of FVS; (2) to evaluate the degree of damage caused by the FVS on the permeability; and (3) to discuss the output mechanism and prevention measures of coal fines.

## Geological Setting

The Junggar Basin is located in the northern Xinjiang Uygur Autonomous Region, northwest China, and is the second largest inland basin in China. The basin covers an area of 13 × 10^4^ km^2^; it is 370 km from north to south and 700 km from east to west.

**Figure 1:**
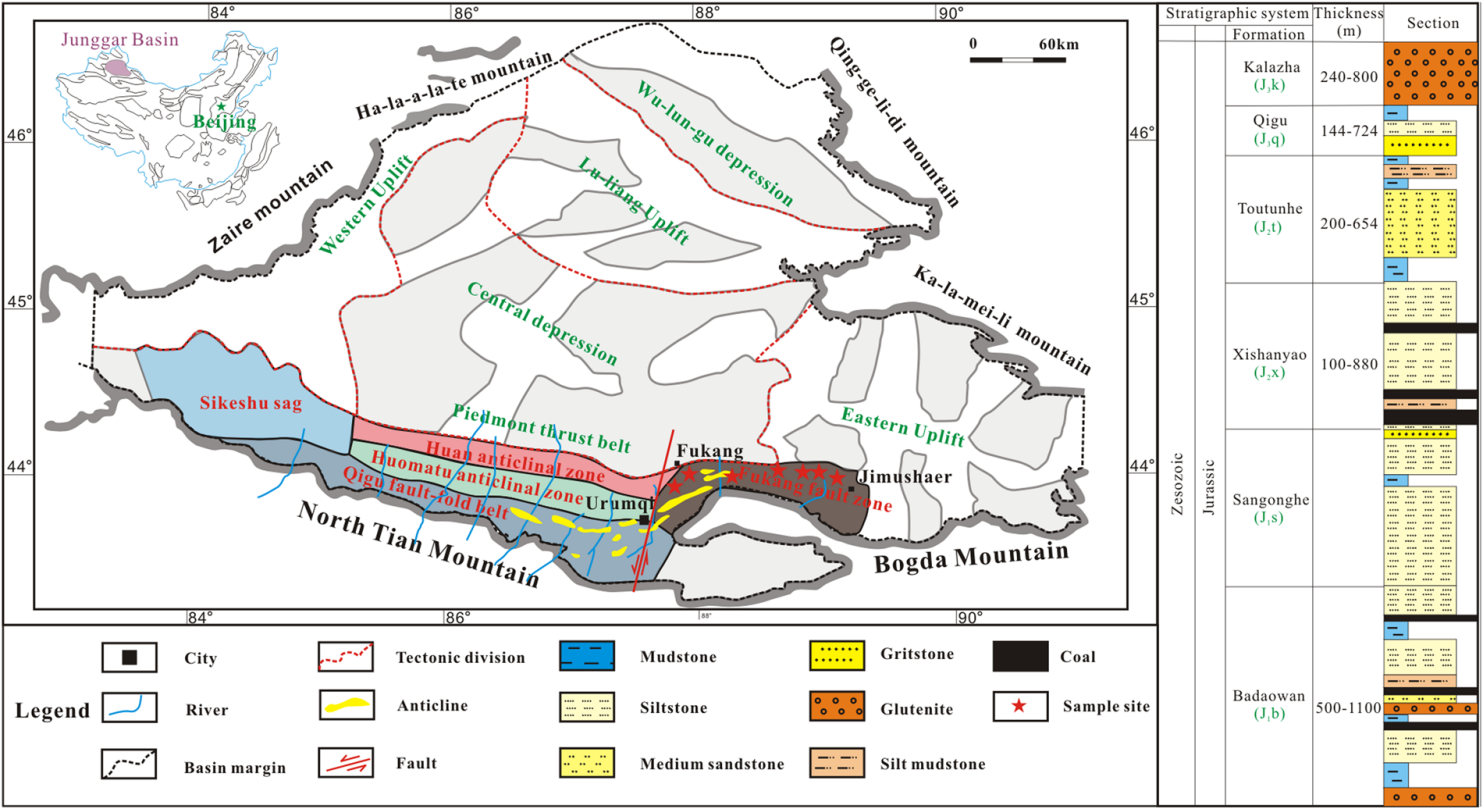
Map showing the geographical position of the tectonic units and the stratigraphic column for the coal-bearing strata in the southern Junggar Basin (modified from^26^ ^(I am a co-author)^).

The southern Junggar Basin belongs to the piedmont thrust belt of the North Tianshan Mountain and has undergone Hercynian, Indosinian, Yanshan, and Himalayan tectonic movement from the late Paleozoic to the Quaternary. The North Tianshan Mountain sits in a multi-phase superimposed inheritance structural belt. The Junggar Basin was formed in the late Hercynian foreland basin and is generally divided into 5 secondary structure units (i.e., the Sikeshu sag, the Qigu fault-fold belt, the Huomatu anticlinal zone, the Huan anticlinal zone, and the Fukang fault zone) (Fig. 1). The surface of the southern Junggar Basin is mostly covered by Quaternary strata, with partial exposure of the Jurassic strata. The Badaowan Formation of the Lower Jurassic (J_1_b) and the Xishanyao Formation of the Middle Jurassic (J_2_x) are the two main coal-bearing strata, and they are widespread in the basin (Fig. 1).

In recent years, approximately 100 CBM wells have been drilled in the Fukang fault zone to exploit the producing Xishanyao coal-bearing strata. The average gas production is approximately 1500 m^3^ per day, a record for medium-low rank CBM production in China. However, the gas production considerably varies by well. One of the main reasons for this variation is that the geologic structure of this area is complicated, resulting in a regional variation in coal structure that drives the variation in coal fines production during CBM development.

## Methodology

### Basic information of the coal samples

Table 1 shows the basic information of the tested coal cores that were collected from the Xishanyao Formation in the Fukang Block. The coal cores are cylindrical, with lengths between 2.986 and 4.112 cm and diameters between 2.553 and 2.566 cm. The collected samples are low-grade metamorphic coals with a vitrinite reflectance (*R*_*o*_) ranging from 0.70% to 0.78%. The coals have a high vitrinite content (34.36–65.1%), with lesser inertinite contents (14.9–36.83%) and liptinite contents (4.89–11.5%). The porosity and permeability of the coal samples range from 3.685–6.881% and 2.586–6.338 mD, respectively.

**Table 1:** Basic information of the prepared coal cores.

Coal core no.	Porosity (%)	*R*_o_ (%)	Length (cm)	Diameter (cm)	Coal composition (vol.%)				Permeability (mD)	Experimental program
					Vitrinite	Inertinite	Liptinite	Mineral		
CSa0	5.018	0.74	3.421	2.554	52.14	23.21	8.62	16.39	3.693	A
CSa1	3.685	0.78	2.986	2.563	39.35	28.6	10.82	21.23	2.586	B
CSa2	4.098	0.76	4.112	2.557	48.8	30.4	8.71	12.09	2.794	
CSa3	4.421	0.75	3.696	2.566	51.05	24.83	7.7	16.42	3.355	
CSa4	5.136	0.73	3.337	2.553	34.36	33.4	10.53	21.71	3.707	
CSa5	6.523	0.71	3.527	2.565	50.15	20.5	4.89	24.46	5.246	
CSa6	6.568	0.71	3.613	2.561	46.37	36.83	5.85	10.95	5.477	
CSa7	6.881	0.70	3.536	2.562	59.00	20.60	11.50	8.90	6.338	C
CSa8	5.364	0.74	3.341	2.558	65.10	14.90	9.10	10.90	4.283	D
CSa9	5.381	0.74	3.342	2.562	52.20	27.40	6.40	14.00	4.312	

### Experimental setup

**Figure 2:**
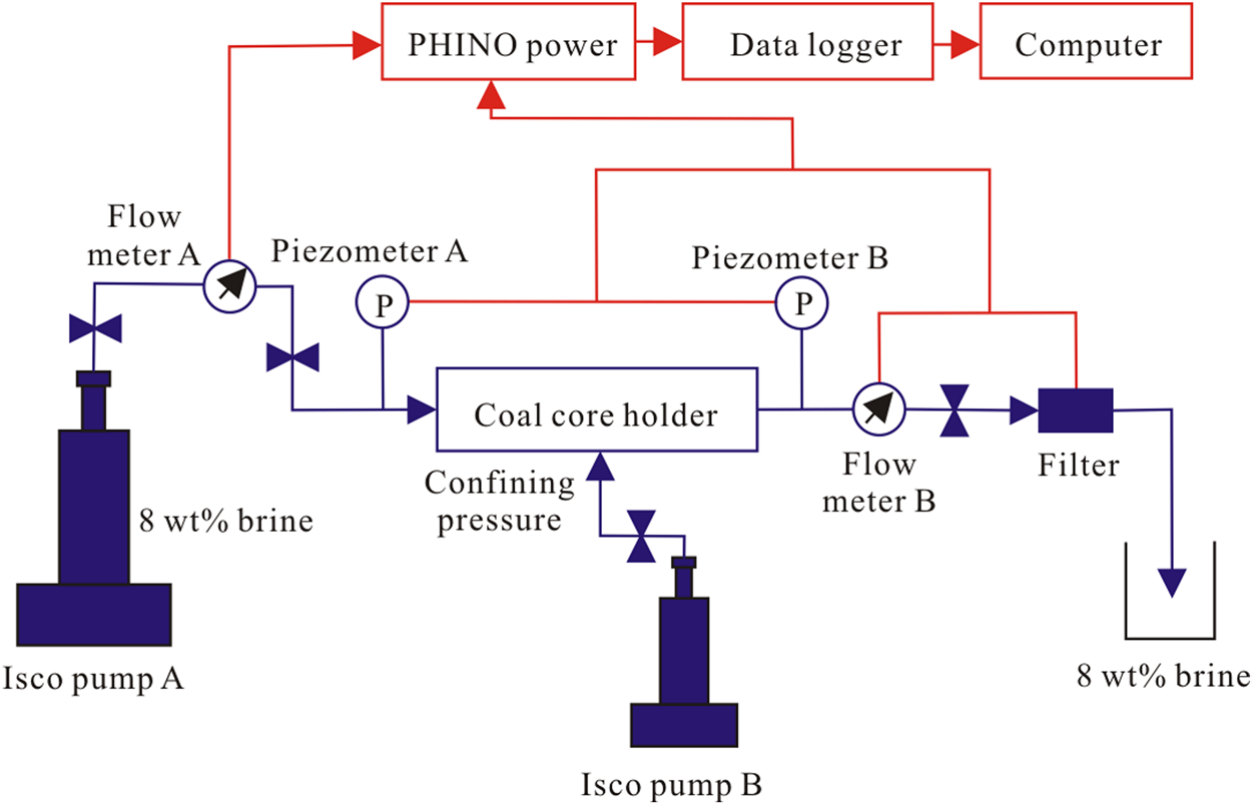
Diagram of the coal core FVS experimental setup.

Figure 2 presents the flow chart of the experimental setup, which is composed of a driving pump (ISCO 1000D Syringe Pump A for brine injection), a coal holder, a data measurement and acquisition system, and a computer calculation system. ISCO 1000D Syringe Pump B provides the confining pressure around the core holder. Piezometers A and B are installed in the inlet and outlet of the core holder, respectively, to measure the real-time injection pressure and outlet pressure. During the core flow experiments, flow meter A and B were installed in the inlet and outlet of the core holder, respectively, to measure the brine flow velocity in real time. A filter was used to collect the coal fines in the outlet fluid. The particle size measurement of the coal fines was analyzed with the Mastersizer 2000, manufactured by the British Malvern Corporation. All experimental protocols were approved by Coal Reservoir Laboratory of National Engineering Research Center of CBM Development & Utilization (Beijing, China).

### Experimental procedure

Four series of experiments were performed to reveal the FVS of coal cores in four programs, each with a different set of experimental conditions.

Program A: Coal core experiments for different confining pressures and a constant fluid velocity.

① The coal cores were put into a drying chamber at a temperature of 110 °C for 12 hours. A device composed of two connected bottles was used to saturate the coal cores. The dried coal cores were put into one bottle, and the standard brine (a mineralization degree of 8% with the mass ratio of NaCl:CaCl_2_:MgCl_2_·6H_2_O = 7.0:0.6:0.4) was placed in the other bottle. The device was vacuumed for 12 hours. Then, the brine was poured into the bottle with the coal cores to immerse them, and the device was vacuumed for another 12 hours.

② The coal cores were placed in the core holder, and the confining pressure was set to 4 MPa.

③ The initial permeability was measured as the brine was injected at a constant flow velocity of 0.01 cm^3^/s.

④ The brine was then injected at a constant flow velocity of 0.05 cm^3^/s, and the corresponding permeability was measured.

⑤ The coal fines were collected in the filter, and the particle size was measured.

⑥ Steps ④ and ⑤ were repeated at different confining pressures: 8 MPa and 10 MPa.

Program B: Coal core experiments with different initial permeabilities and different flow velocities.

① Steps ①, ② and ③ in program A were repeated.

② Steps ④ and ⑤ in program A were repeated at the flow velocities of 0.015, 0.02, 0.03, 0.04, 0.06, 0.08, and 0.1 cm^3^/s.

Program C: Coal core experiments under different injection volumes.

① Steps ①, ② and ③ in program A were repeated.

② A total of 80 cm^3^ of brine was injected at a constant flow velocity of 0.01 cm^3^/s, and the permeability was measured when the injection volume reached 10, 20, 30, 50 and 80 cm^3^.

③ Step ② was repeated at the flow velocities of 0.015, 0.03, 0.05, and 0.08 cm^3^/s.

Program D: Coal core experiments with increasing fluid velocities.

① Two coal cores with similar initial permeabilities were prepared from the same coal sample.

② Steps ①, ② and ③ in program A were repeated.

③ For one coal core, 80 cm^3^ of brine was injected at gradually increasing flow velocities of 0.01, 0.02, 0.05, and 0.1 cm^3^/s (20 cm^3^ brine was injected at each fluid velocity).

④ The total output of the coal fines and the permeability of the coal core were measured and recorded at each flow velocity.

⑤ For the other coal core, 80 cm^3^ of brine was injected at quickly increasing flow velocities (i.e., from 0 to 0.1 cm^3^/s).

⑥ Step ④ was repeated.

### Permeability calculation

Newtonian fluid flow through porous media follows Darcy’s law^22^:

1 $$Q=-86.4\frac{kA({p}_{A}-{p}_{B})}{\mu L}$$

where *k* is the permeability, μm^2^, 1 μm^2^ = 1 D = 10^3^ mD; *p*_*A*_ is the injection pressure, MPa; *p*_*B*_ is the outlet pressure, MPa; *A* is the cross-sectional area of the coal core, m^2^, 1 m^2^ = 10^4^ cm^2^; *µ* is the brine viscosity, mPa·s; *L* is the length of the coal core, m, 1 m = 100 cm; and *Q* is the outlet flow velocity, which is measured by flow meter B, m^3^/s, 1 m^3^/s = 10^6^ cm^3^/s.

The coal core permeability was calculated 3 times using Formula (1) when the value of *P*_*A*_ − *P*_*B*_ was consistent for more than 10 min, and the relative error was less than 3%.

### Permeability damage and velocity

The permeability damage rate is determined by Eq. (2) ^23^:

2 $${D}_{{\rm{ki}}}=\frac{{k}_{0}-{k}_{{\rm{i}}}}{{k}_{0}}\times 100 \% $$

where *D*_*ki*_ is the damage rate of the permeability, %; *k*_0_ is the initial permeability of the coal core, mD; and *k*_i_ is the permeability measured under different flow velocities, mD.

## Results and Discussion

### Coal fines output under different confining pressures

The permeability variation in the coal core at different confining pressures according to the experimental program A is shown in Table 2. At the confining pressure of 4 MPa, the output of the coal fines is high, and the coal permeability slightly decreases. When the confining pressure increases to 8 MPa, the output of the coal fines is reduced, and the coal permeability rapidly decreases. At the confining pressure of 12 MPa, the output of coal fines is only 0.4 mg, and the coal permeability decreases to 2.521 mD. The observed permeability increase is an experimental phenomenon due to the small extrusion force acting on the coal core. The coal fines in the fractures can be easily produced at a low confining pressure, which can improve the coal core permeability. However, the migration of coal fines also causes FVS in the coal core; thus, a small decline in coal permeability (0.122 mD) occurred at the confining pressure of 4 MPa. However, if the confining pressure continues to increase, the extrusion force acting on the coal core also increases, leading to an increase in the effective stress and a decrease in the fracture apertures. When the driving flow velocity is constant, less coal fines are produced with the fluid, and the coal permeability decreases as a result.

**Table 2:** Permeability variation and coal fines output at different confining pressures and a constant flow velocity.

Coal core no.	CSa0		
Confining pressure (MPa)	4	8	12
Initial permeability (mD)	3.693		
Current permeability (mD)	3.571	2.863	2.521
Total mass of the coal fines at the outlet (mg)	4.8	6.9	7.3

**Figure 3:**
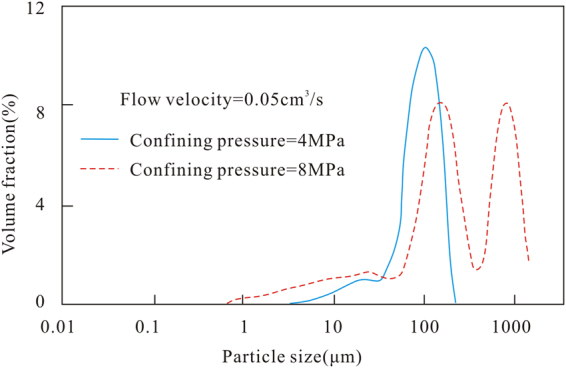
Particle size distribution curves of the coal fines at different confining pressures and a constant flow velocity.

Figure 3 shows that the particle size distribution curves of the coal fines produced during the experiments with confining pressures of 4 MPa and 8 MPa are bimodal. However, only one major peak appears between 50 and 180 μm at the confining pressure of 4 MPa, while major peaks appear in the two ranges of 190–260 μm and 550–1040 μm at the confining pressure of 8 MPa. Hence, the particle size of the produced coal fines is generally larger at 8 MPa than at 4 MPa. Despite the reduction in the output of coal fines with the increase in confining pressure, the extrusion force on the coal core gradually increases, which leads to a higher particle size concentration of larger produced coal fines.

### Coal fines output and permeability variations at different flow velocities

**Figure 4:**
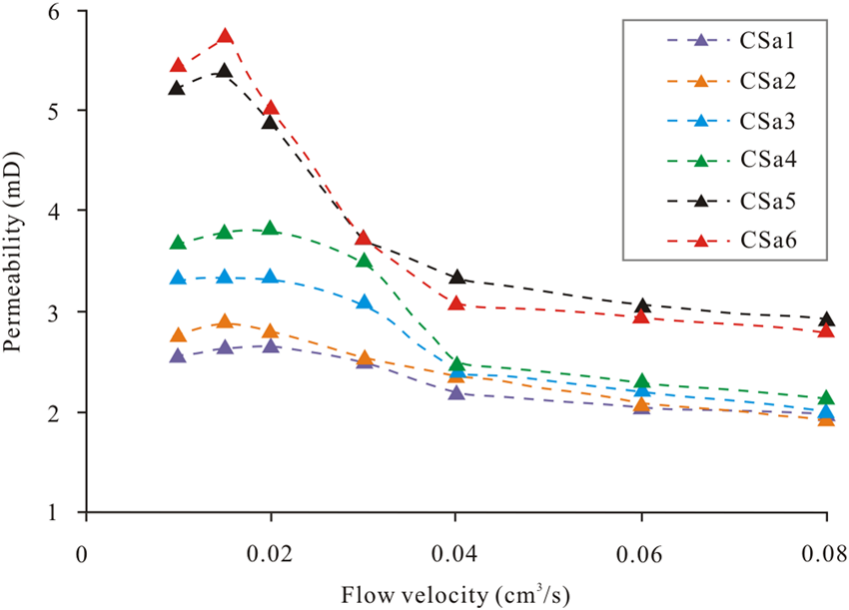
Relationship between the flow velocity and coal core permeability.

Table 3lists the permeability variation in the coal cores at different flow velocities, according to the experimental program B. The permeability of the 6 coal cores remains constant, and no coal fines are produced at the flow velocity of 0.01 cm^3^/s. When the flow velocity is increased to 0.015 cm^3^/s, a small amount of coal fines are produced, and the permeabilities of all 6 coal cores increase slightly. As the flow velocity is increased to 0.02 cm^3^/s, the permeabilities of cores CSa1, CSa2, CSa3 and CSa4 are still slightly higher than their initial values. However, the permeabilities of CSa5 and CSa6, which have relatively high initial permeabilities, decline significantly, indicating that the FVS effect occurs in CSa5 and CSa6 at the flow velocity of 0.02 cm^3^/s. With a further increase in the flow velocity (over 0.03 cm^3^/s), the FVS effect occurs in all the coal cores, accompanied by the reduction in permeability (Fig. 4). The permeability decreases slowly when the flow velocity is increased to 0.04 cm^3^/s. As seen in Table 3, the decrease in the coal permeability ranges from 0.379 to 2.372 mD (mean of 1.2 mD) when the flow velocity increases from 0.01 to 0.04 cm^3^/s, and decrease in the permeability ranges from 0.228 to 0.476 mD (mean of 0.391 mD) when the flow velocity increases from 0.04 to 1.0 cm^3^/s. Hence, generation of coal fines should be strictly controlled to alleviate FVS of coal permeability during the early stage of CBM drainage.

**Table 3:** Permeability and its maximum damage rate at different flow velocities.

Coal core no.		CSa1	CSa2	CSa3	CSa4	CSa5	CSa6
Initial permeability (mD)		2.586	2.794	3.355	3.707	5.246	5.477
Permeability under different flow velocities (mD)	0.01 (cm^3^/s)	2.586	2.794	3.355	3.707	5.246	5.477
	0.015 (cm^3^/s)	2.66	2.924	3.361	3.807	5.419	5.765
	0.02 (cm^3^/s)	2.679	2.829	3.363	3.841	4.904	5.052
	0.03 (cm^3^/s)	2.516	2.556	3.126	3.537	3.738	3.758
	0.04 (cm^3^/s)	2.207	2.376	2.419	2.501	3.344	3.105
	0.06 (cm^3^/s)	2.068	2.102	2.233	2.312	3.077	2.964
	0.08 (cm^3^/s)	2.004	1.951	2.027	2.159	2.941	2.826
	0.1 (cm^3^/s)	1.979	1.900	1.992	2.136	2.909	2.691
Permeability decrease under different flow velocity ranges (mD)	0.01–0.04 (cm^3^/s)	0.379	0.418	0.936	1.206	1.902	2.372
	0.04–0.1 (cm^3^/s)	0.228	0.476	0.427	0.365	0.435	0.414
Maximum rate of permeability damage (%)	23.47	32	40.63	42.38	44.55	50.87

**Figure 5:**
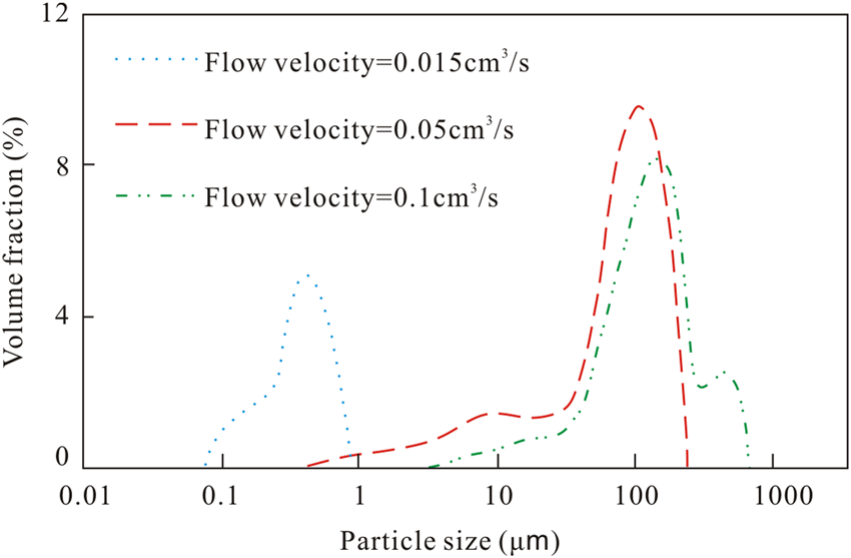
Particle size distribution at different flow velocities.

Figure 5 shows the diameter of the coal fines collected from the outlet as a function of the flow velocities. Some very small coal fines, with a diameter in the range of 0.08–0.9 μm, are produced from the coal cores at the flow velocity of 0.015 cm^3^/s, indicating that the very small coal fines can easily flow through the pore system of a coal core at a low fluid flow velocity, increasing the coal core permeability. In this scenario, the higher the initial permeability is, the greater the permeability increase (Table 3). As the flow velocity increases, larger coal fines are able to migrate. However, larger coal fines cannot flow through pore throats that are smaller than their largest diameter. In addition, the coal fines accumulate in the small pore throats, blocking seepage channels and reducing the coal core permeability. The particle size distribution curve of the coal fines is bimodal for the flow velocities of 0.05 cm^3^/s and 0.1 cm^3^/s. However, the main peak is in the range of 50–190 μm for the flow velocity of 0.05 cm^3^/s, while it is between 70 and 210 μm for the flow velocity of 0.1 cm^3^/s.

Therefore, the critical flow velocity for the occurrence of the FVS effect in the 6 tested coal cores is approximately 0.015 cm^3^/s-0.02 cm^3^/s, and the maximum permeability damage rate caused by the FVS varies from 23.47% to 50.87%. The higher the initial permeability is, the greater the permeability reduction, and the lower the critical flow velocity for the occurrence of FVS. With an increasing displacement velocity, the particle size of the produced coal fines increases, and more coal fines are produced. This result demonstrates that high-intensity drainage during the production of CBM wells may cause greater erosion damage of the coal reservoirs and may lead to the generation of more coal fines of larger sizes.

### Permeability variation at different flow velocities and different injection volumes

**Figure 6:**
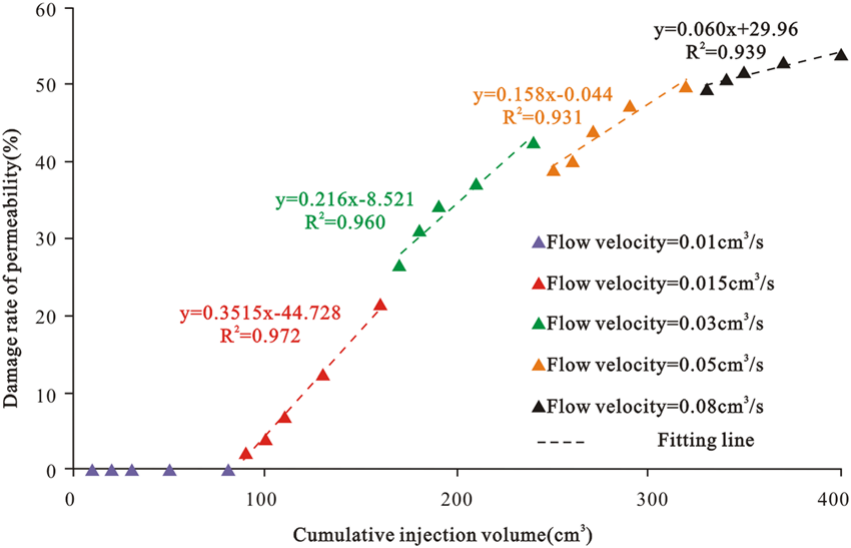
Relationship between the cumulative injection volume and the permeability damage rate at different flow velocities.

Table 4 and Fig. 6 illustrate that the coal core permeability damage rate increases with the flow velocity under a constant injection volume (80 cm^3^). Therefore, the relatively low drainage rate can reduce the reservoir permeability damage due to coal fines. For example, the maximum permeability damage rate of core CSa7 at a flow velocity of 0.015 cm^3^/s is 21.62%, whereas that at a flow velocity of 0.08 cm^3^/s reaches 54.01% under the same total injection volume. Meanwhile, the permeability damage rate has an approximately linear relationship with the flow velocity. At a constant flow velocity, an increase in the cumulative injection volume gradually increases the damage degree. Nevertheless, the overall rate of increase in the damage degree decreases. The permeability damage rate decreases from 1.37 to 0.264 mD when the flow velocity increases from 0.015 to 0.08 cm^3^/s, indicating that the permeability damage mainly occurs in the initial period of the coal fines migration (under a low flow velocity)^24^.

**Table 4:** Permeability damage rate at different flow velocities and different injection volumes.

Coal core no.	Initial permeability (mD)	Flow velocity (cm^3^/s)	Cumulative injection volume (cm^3^)	Current permeability (mD)	Loss of permeability (mD)	Damage rate of permeability (%)
CSa7	6.338	0.01	10	6.203	0	0
			20	6.081		0
			30	5.902		0
			50	5.556		0
			80	4.968		0
		0.015	90	6.203	1.37	2.13
			100	6.081		4.05
			110	5.902		6.88
			130	5.556		12.34
			160	4.968		21.62
		0.03	170	4.651	1.341	26.62
			180	4.372		31.02
			190	4.175		34.13
			210	3.976		37.27
			240	3.627		42.77
		0.05	250	3.864	0.448	39.03
			260	3.784		40.30
			270	3.537		44.19
			290	3.338		47.33
			320	3.179		49.84
		0.08	330	3.196	0.264	49.57
			340	3.128		50.65
			350	3.053		51.83
			370	2.991		52.81
			400	2.915		54.01

According to the experiments of program B and C, in the early stage of CBM development, before gas desorption, small coal fines can be easily produced with flowing water. If the drainage rate is too fast, the large producing pressure drop will lead to the increase in stress around the wellbore and shear brittle failure of the coal. Therefore, a large volume of coal fines is produced, and the FVS effect extends, weakening the seepage capacity of the coal reservoirs and decreasing the gas production capacity of the CBM wells. Therefore, the generation of coal fines should be strictly controlled during the early stage of water drainage.

### Coal fines output and permeability variation with different approaches for increasing the flow velocity

Table 5 lists the permeability damage rate and the coal fine output with different approaches for increasing the flow velocity according to the experimental program D. Two coal cores with similar initial permeabilities show a considerable difference in permeability damage rate due to the difference in the approaches used for increasing the flow rate; compared with the more gradual increase in the flow velocity, the abrupt increase caused greater damage to the coal sample and more coal fines to be generated. The coal cores undergo relatively stable erosion when the displacement flow velocity is slowly increased. However, when the displacement flow velocity increases abruptly, the relatively stable state is disrupted, and the coal body structure may lose stability due to the rapid washing effect of the fluid, resulting the generation of a large amount of coal fines. Therefore, increasing the drainage rate slowly during CBM production may help to control the generation of coal fines and alleviate the reservoir damage caused by FVS. In contrast, surging conditions (such as discontinuous production) will generate more coal fines and more intense damage to the coal reservoirs.

**Table 5:** Permeability damage rate with different approaches for increasing the flow velocity.

Coal core no.	Initial permeability (mD)	Flow velocity (cm^3^/s)	Maximum damage rate of permeability (%)	Total mass of coal fines from the outlet (mg)
CSa8	4.283	0 → 0.01 → 0.02 → 0.05 → 0.1	44.38	10.15
CSa9	4.312	0 → 0.1	59.26	19.18

During drainage of the CBM wells, some wells are shut-in for blocking removal because of the high rate of coal fines output and the adjustment of the development plan. These wells may produce a large amount of coal fines when they are reopened. The coal fines result from the fast reservoir water flow during treatments. Since the working fluid level raises during shut-in, the coal seams are saturated in water, decreasing the coal strength. When the shear stress acting on the coal matrix is greater than the tensile strength of the coal, shear failure will occur within the coal, and more coal fines will be generated. After the CBM wells are reopened for production, reservoir water cannot efficiently carry coal fines due to its low energy. If the produced coal fines cannot freely discharge, they will amalgamate in the pore throats, blocking the gas production paths and decreasing the gas yield.

### Mechanism of permeability change caused by the FVS

The relationship between the permeability and pore throat radius in porous media can be expressed by the Carman-Koreny formula^25^.

3 $$k=\frac{\varphi {r}^{2}}{8{\tau }^{2}}$$

where *k* is the permeability, μm^2^, 1 μm^2^ = 1 D = 10^3^ mD; *ϕ* is the porosity, %; *r* is the throat radius, μm; and *τ* is the degree of tortuosity, which characterizes the convolution of the fluid flow pathway. The shape of the pores and pore throats can also affect the experimental results. In addition, the composition of the coal and the variations in the industrial components and macerals will lead to some uncertainties in the experiment.

By using Formula (3), the coal reservoir permeability was determined to be positively correlated with the throat radius. It can be seen from the above experiments that the migration and output of the coal fines within the coal is a complex process affected by many factors, such as the reservoir pressure, coal initial permeability, injection volume and flow velocity. Some of these factors can decrease the fracture aperture, and some of them can generate large fines that narrow the pore throat radii. In either case, the generation of coal fines would decrease the permeability of the coal reservoir. With more coal fines in the reservoir, when the coal fines migrate with the water, the migration channels are more easily blocked.

**Figure 7:**
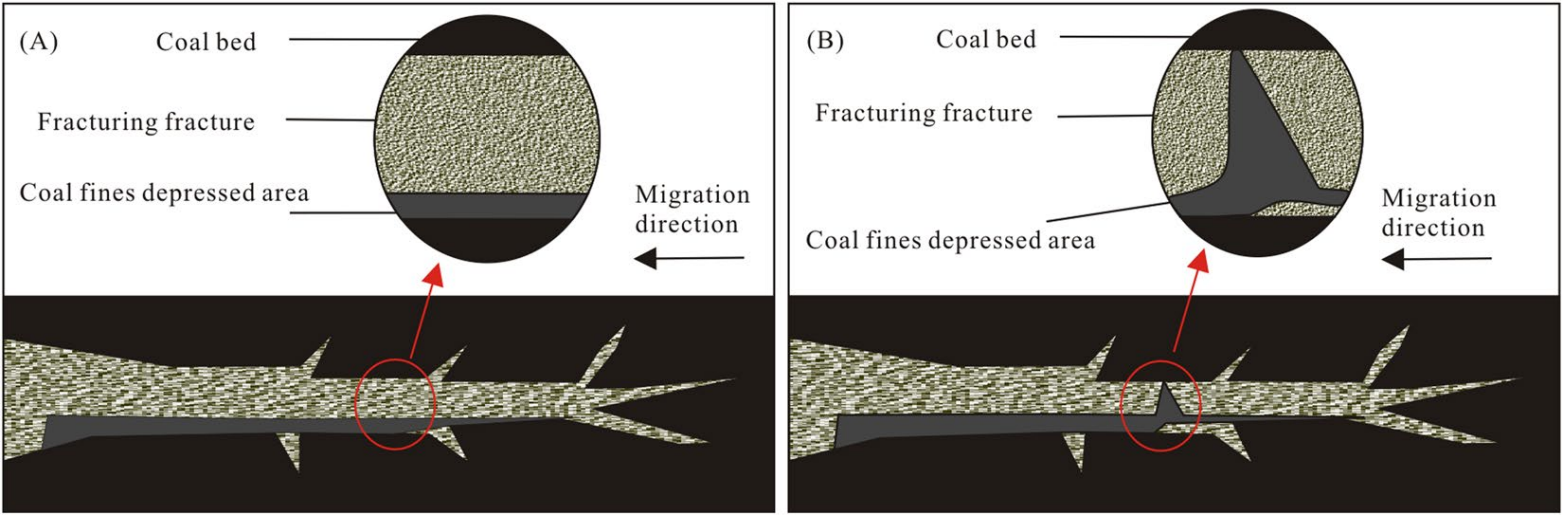
Illustrative diagram of coal fine migration during the drainage process of CBM wells.

Normally, the coal fines migrate with water along large fractures, enter the wellbore and discharge via the pump, either suspended in the water or falling to the bottom of the fluid pathway during migration. However, if the drainage speed increases too quickly, the fractures near the wellbore are narrowed due to the increase in effective stress; simultaneously, some larger coal fines may be transported due to the increase in flow velocity. When the coal fines arrive at narrow areas of the fractures, the flow velocity in front of the coal fine slows down, but that behind the particle does not. Under this velocity difference, the coal fines can easily block the seepage paths (Fig. 7).

Therefore, to prevent the production of a large amount of coal fines, which are caused by the pressure surging from the abrupt increase in the drainage speed, the working fluid level of CBM wells should be decreased slowly, continuously and stably during the drainage process. Before gas production, the drainage rate should be slow and stable to avoid an abrupt change in the effective stress of the coal matrix, which may be induced by the abrupt increase or decrease in the working fluid level, leading to the generation of coal fines. Meanwhile, the drainage process should be continuous because if the fluid flow stops, coal fines will be deposited in fractures, equipment surfaces and wellbores. Additionally, the pump type and efficiency should be optimized and improved according to the volume of the drainage liquid and the amount of carried coal fines. After gas production, the casing pressure should be controlled to avoid the collapse and blocking of coal seams caused by pressure surging.

## Conclusions

This study evaluated the effect of FVS on the coal fines output and permeability damage of coal cores. The major conclusions are summarized as follows:

1. At an equal flow rate, the coal permeability decreases sharply with increasing confining pressure for two reasons: the increase in effective stress compresses the pore-fracture systems, and a strong FVS effect occurs due to the generation of large coal fines.
2. The critical flow velocity for the initiation of the FVS effect ranges between 0.015 cm^3^/s and 0.02 cm^3^/s in the 6 coal cores tested. The greater the initial permeability is, the more intense the damage due to the FVS effect, and the lower the critical flow velocity. As the flow rate increases, the generated coal fines gradually increase in particle diameter size.
3. With increasing flow velocity and injection volume, the permeability damage rate increases, but the rate of increase in the damage decreases, indicating that the damage to permeability due to FVS mainly occurs in the early stage of coal fines migration. Furthermore, an abrupt increase in the flow velocity can damage reservoirs and generate more coal fines.
4. On one hand, the production of the coal fines is beneficial for the formation of the gas-water seepage path; on the other hand, it may inhibit the gas-water flow and decrease the gas yield if too many coal fines are generated and if the generation rate is too fast.

### Data availability statement

The datasets generated during and/or analysed during the current study are available from the corresponding author Shu Tao on reasonable request.
